# Reconstitution of the membrane protein OmpF into biomimetic block copolymer–phospholipid hybrid membranes

**DOI:** 10.3762/bjnano.7.80

**Published:** 2016-06-21

**Authors:** Matthias Bieligmeyer, Franjo Artukovic, Stephan Nussberger, Thomas Hirth, Thomas Schiestel, Michaela Müller

**Affiliations:** 1 Institute of Interfacial Process Engineering and Plasma Technology, Department of Chemical Interfacial Process Engineering, University of Stuttgart, Nobelstraße 12, 70569 Stuttgart, Germany; 2 Institute of Biomaterials and Biomolecular Systems, Department of Biophysics, University of Stuttgart, Pfaffenwaldring 57, 70550 Stuttgart, Germany; 3 Fraunhofer Institute for Interfacial Engineering and Biotechnology, Department of Interfacial Engineering and Materials Science, Nobelstraße 12, 70569 Stuttgart, Germany

**Keywords:** biomimetics, block copolymer, lipopolymer mixture, OmpF reconstitution, self-assembly

## Abstract

Structure and function of many transmembrane proteins are affected by their environment. In this respect, reconstitution of a membrane protein into a biomimetic polymer membrane can alter its function. To overcome this problem we used membranes formed by poly(1,4-isoprene-*block*-ethylene oxide) block copolymers blended with 1,2-diphytanoyl-*sn*-glycero-3-phosphocholine. By reconstituting the outer membrane protein OmpF from *Escherichia coli* into these membranes, we demonstrate functionality of this protein in biomimetic lipopolymer membranes, independent of the molecular weight of the block copolymers. At low voltages, the channel conductance of OmpF in 1 M KCl was around 2.3 nS. In line with these experiments, integration of OmpF was also revealed by impedance spectroscopy. Our results indicate that blending synthetic polymer membranes with phospholipids allows for the reconstitution of transmembrane proteins under preservation of protein function, independent of the membrane thickness.

## Introduction

Protein pores facilitate the transfer of molecules across lipid membranes with high fidelity and selectivity. In the field of biotechnology, they are particularly attractive for single-molecule DNA sequencing [1–5] and stochastic sensing of ions and macromolecules [6–10]. The well-defined dimensions of the protein pores furthermore offer a high potential in technical applications, for example the desalination of water using aquaporins or the controlled release of antibiotics from OmpF-functionalized nanoreactors [11–15].

Although the chemical and physical properties of natural phospholipid bilayers are optimized for the functional reconstitution of integral proteins, these membranes are often not applicable in technical processes due to their high fluidity and lack of long-term stability [16–17]. Therefore, novel approaches substitute phospholipids by amphiphilic block polymers that form membranes with a mechanical strength up to 10 times larger than that of phospholipid bilayers [18–24]. Vesicles formed by synthetic block polymers (polymersomes) can be modified in a similar way as vesicles made from phospholipids (liposomes) [25–28]. Although polymer membranes are generally thicker than those made of lipids, the successful integration of membrane proteins has been shown [24,29–32]. However, their function and dynamics could be impaired due to the unnatural environment within the polymer membranes [33–37]. Bacterial OmpF channels, for example, did not show typical closing behavior when reconstituted into poly(2-methyl-2-oxazoline-*block*-dimethylsiloxan-*block*-2-methyl-2-oxazoline) triblock copolymer membranes [38].

More recently, it has been proposed to ‘tune’ the properties of polymeric membranes by blending them with phospholipids to bring together the mechanical stability and the inherent biofunctionality of polymeric and lipidic membranes, respectively [39–40]. Until now, most studies focused on the conditions influencing the appearance of lipopolymer assemblies, such as temperature or composition details [41–44]. Also the influence of additives, e.g., detergents, oligosaccharides or enzymes on polymer–lipid miscibility and membrane stability has been analyzed [45]. Although the insertion and distribution of OmpF and the potassium channel MloK1 in lipopolymer films has been studied, the functionality of both proteins in a lipopolymer environment has not been assessed yet [46–47].

In the present work, we studied the reconstitution of OmpF from *Escherichia coli* into biomimetic lipopolymer membranes, generated by self-assembly of amphiphilic poly(1,4-isoprene-*block*-ethylene oxide) block copolymers (PIPEO) and 1,2-diphytanoyl-*sn*-glycero-3-phosphocholine (DPhPC). To vary the membrane thickness, PIPEO with three different molecular weights were chosen from a library of various PIPEO, which we had synthesized in an attempt to design new amphiphilic molecules that self-assemble in aqueous solutions into membranes (unpublished results).

PIPEO was used because it possesses a low glass-transition temperature and thus high chain flexibility mandatory for protein incorporation [31]. In contrast to the mostly used hydrophobic poly(dimethylsiloxane) blocks, the structure of the hydrophobic poly(isoprene) blocks of PIPEO resembles more closely that of unsaturated hydrophobic lipid tails [30]. Moreover, PIPEO can potentially be cross-linked within the hydrophobic block to further increase membrane stability.

OmpF was chosen as transmembrane protein, because it remains stable not only in harsh detergents but also at high temperatures [48–50]. In order to retain the polymeric character of the membranes, the amount of lipid used was limited to 10 mol %. Eventually, membranes were analyzed using confocal laser scanning microscopy, voltage clamp measurements and impedance spectroscopy.

## Results and Discussion

### Block copolymer synthesis

**Figure 1:**
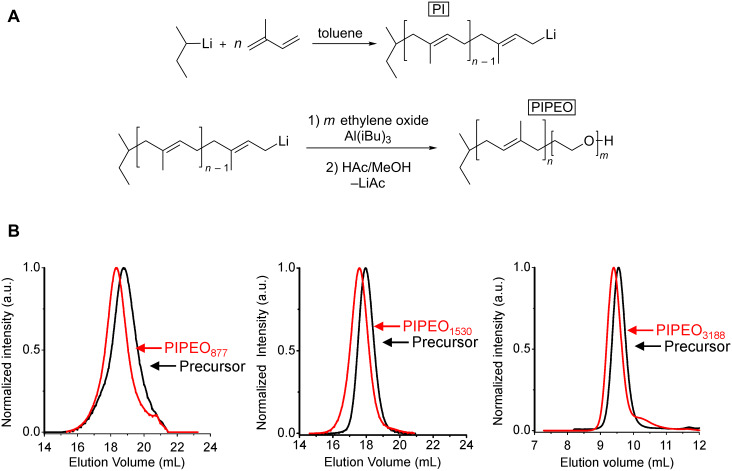
Synthesis of PIPEO block copolymers. (A) Reaction scheme for the synthesis of poly(1,4-isoprene-*block*-ethylene oxide) block copolymers. Anionic polymerization of isoprene in toluene was initiated with *sec*-butyl lithium. Sequential polymerization of ethylene oxide was enabled by triisobutyl aluminium. (B) Size exclusion chromatography traces of synthesized PIPEO block copolymers and corresponding poly(isoprene) precursors. The shifts towards lower elution volumes of the block copolymers indicate higher molecular weights.

Poly(1,4-isoprene-*block*-ethylene oxide) block copolymers (PIPEO) were designed at poly(isoprene) (PI) to poly(ethylene oxide) (PEO) ratios that are known to foster the formation of polymeric bilayers in aqueous solutions [19–20,51]. The three copolymers PIPEO_877_ (**1**), PIPEO_1530_ (**2**) and PIPEO_3188_ (**3**) were synthesized with molecular weights between 877 g mol^−1^ and 3188 g mol^−1^ to allow for a compromise between membrane fluidity, stability and thickness (Figure 1A) [18,21]. The molecular properties were analyzed by size-exclusion chromatography (Figure 1B) and ^1^H NMR (Supporting Information File 1, Figure S1).

According to anionic polymerization theory, the amount of 1,4-isoprene units in PIPEOs was larger than 88% due to the polymerization in toluene and the polydispersity indices (PDI) being relatively low (Table 1). Only PIPEO_877_ showed an increased polydispersity index, which was likely caused by the high amount of *sec*-butyllithium used as initiator to obtain the low molecular weight of the polymer.

**Table 1:** Molecular properties of PIPEO block copolymers used in this study.^a^

block copolymer	molecular weight^b^ (g/mol)	fraction of PI^b^ (w/w)	polydispersity index of PI^b^	polydispersity index of PIPEO^c^

PIPEO_877_	877	0.70	1.28	1.32
PIPEO_1530_	1530	0.71	1.09	1.16
PIPEO_3188_	3188	0.64	1.07	1.11

^a^PI: poly(1,4-isoprene) block; PIPEO: poly(1,4 isoprene-*block*-ethylene oxide) block copolymer; ^b^determined by ^1^H NMR spectroscopy; ^c^determined by size-exclusion chromatography.

The polydispersity indices of the PIPEO block copolymers were slightly larger than those of the PI precursors, probably because of side reactions during the polymerization of ethylene oxide utilizing the monomer-activated approach [52]. However, an incomplete initiation of the second block could be excluded as side reaction since no tailing within the size exclusion chromatography traces has been observed. Thus, effects on the self-assembly behavior of the block copolymers in aqueous solutions were not expected.

### PIPEO block copolymer–phospholipid membranes

**Figure 2:**
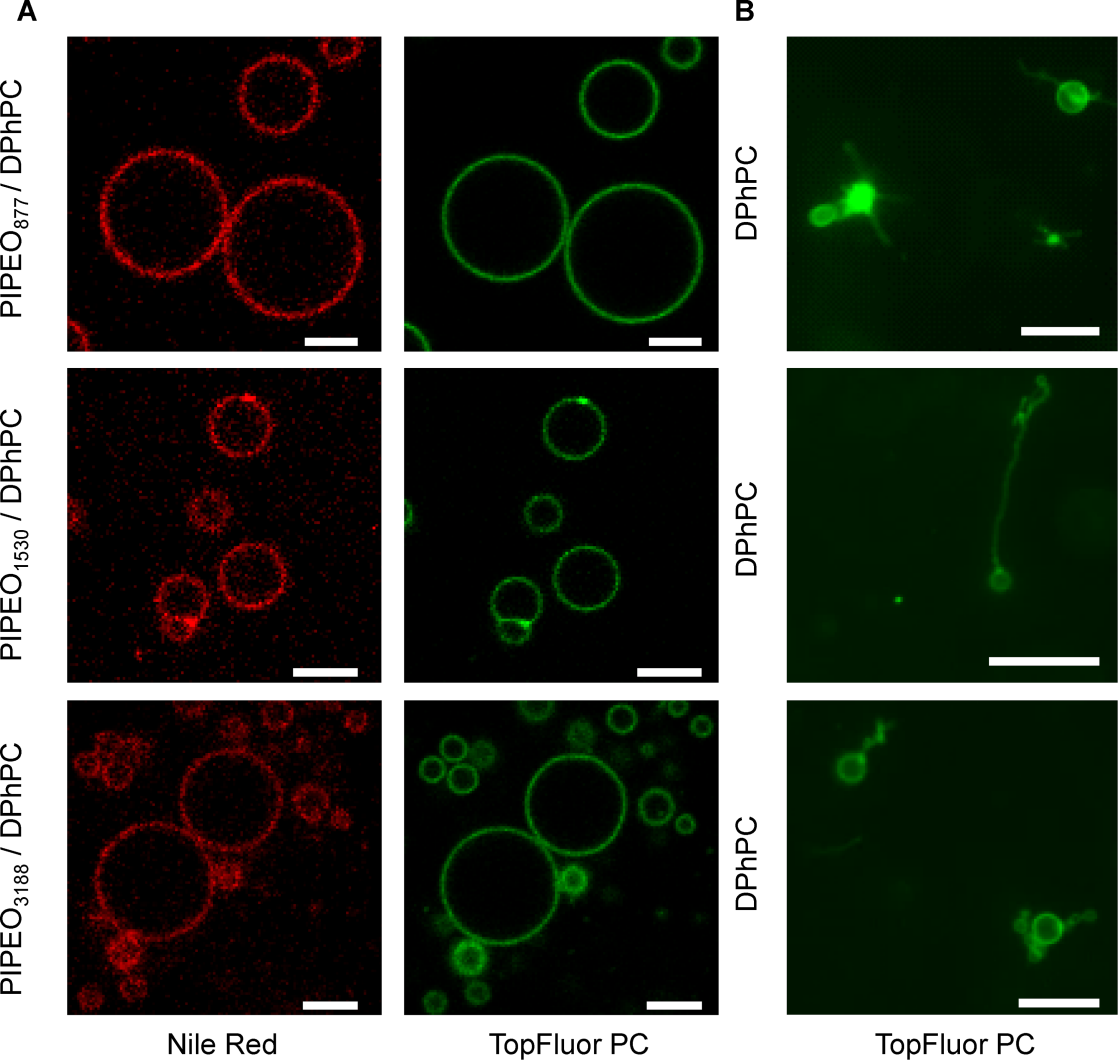
Confocal images of (A) giant unilamellar vesicles created by electroformation in saccharose solution from lipopolymer mixtures comprising 90 mol % PIPEO block copolymers and 10 mol % phospholipids and (B) vesicles obtained from pure DPhPC with the same electroformation parameters as for the lipopolymer mixtures shown in A. Vesicular membranes were fluorescently labeled with TopFluorPC and nile red. The scale bars correspond to 20 µm.

As mixtures of PIPEO and DPhPC have not been analyzed before, the ability of this type of lipopolymer mixture to form membranes, as well as the appearance and the characteristics of these membranes were examined. To assess the miscibility of PIPEO and DPhPC, block copolymer–phospholipid vesicles (lipopolymersomes) were prepared by electroformation [53] in aqueous solutions. Confocal fluorescence microscopy of vesicles dyed with fluorescent lipid (TopFluorPC) and nile red showed that blends of 90 mol % PIPEO block copolymers and 10 mol % DPhPC form giant vesicles (Figure 2A). Nile red was used as a general hydrophobic dye to visualize the membranes; TopFluorPC was used to assess possible phase separation between lipids and polymers. The molecular weight of PIPEO appeared to have no observable effect on the formation of vesicles despite the hydrophobic mismatch between lipid and polymer. This finding indicated that the selected PIPEO and DPhPC were highly miscible. The absence of phase separation towards micrometer-scale lipid- and polymer-rich domains was in line with other lipopolymer mixtures at a similar lipid-to-polymer ratio [39,42,45] and similar membrane curvature [39–41,43–45,54–55]. Lipopolymer vesicles formed from mixtures containing more than 50 mol % DPhPC, however, revealed a heterogeneous distribution of TopFluorPC and a clear phase separation between polymer and lipid (Supporting Information File 1, Figure S2). Pure DPhPC vesicles prepared under similar conditions were significantly smaller than those composed of PIPEO and DPhPC (Figure 2B). They revealed completely different morphologies than the lipopolymersomes.

Using impedance spectroscopy, we attempted to gain insight into the electrical properties of planar PIPEO/DPhPC lipopolymer membranes. As reference, pure PIPEO (Supporting Information File 1, Figure S3) and DPhPC membranes were characterized as well. The spectra were interpreted in terms of an equivalent circuit consisting of an ohmic resistance in series with a resistance–capacitance (RC) pair in parallel, corresponding to an electrolyte–membrane–electrolyte interface [16,56] (Table 2).

**Table 2:** Electrochemical properties of lipopolymer membranes and bilayers from pure amphiphiles.

membrane composition	membrane resistance^a^ (GΩ)	membrane capacitance^a^ (pF)

PIPEO_877_/DPhPC	1.1 ± 0.2	50.3 ± 2.5
PIPEO_1530_/DPhPC	13.9 ± 7.5	50.4 ± 0.8
PIPEO_3188_/DPhPC	0.2 ± 0.1	47.5 ± 2.1
PIPEO_877_	1.2 ± 0.4	41.8 ± 8.9
PIPEO_1530_	2.8 ± 0.2	49.9 ± 0.8
PIPEO_3188_	1.0 ± 0.3	43.5 ± 5.0
DPhPC	0.5 ± 0.2	47.2 ± 4.0

^a^All membrane resistance *R*_m_ and membrane capacitance *C*_m_ values represent an average of *N* = 10 independent experiments. The measured membrane area was approx. 5000 µm^2^.

The membranes formed by PIPEO_877_/DPhPC, PIPEO_1530_/DPhPC, PIPEO_877_ and PIPEO_1530_ revealed ohmic resistances above 1 GΩ. PIPEO_3188_ yielded a resistance close to 1 GΩ. In contrast, the mean resistance of membranes composed of PIPEO_3188_/DPhPC was significantly lower. Nevertheless, the resistances of all the lipopolymer membranes studied appeared to be sufficiently high to allow for monitoring single-channel currents across these membranes [57–58].

**Figure 3:**
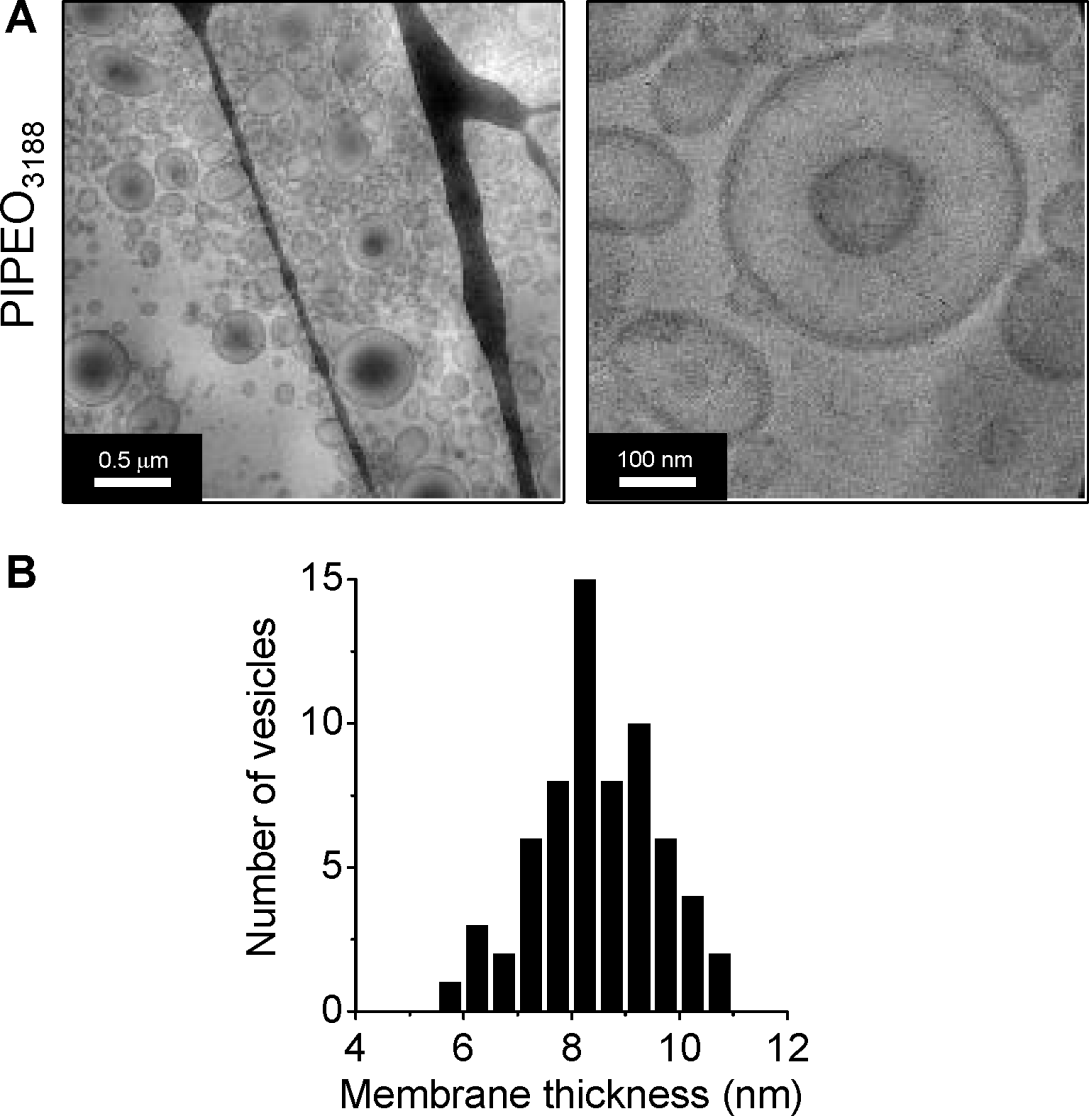
Thickness of pure PIPEO membranes determined by cryo-electron microscopy. (A) Micrographs of polymersomes formed by PIPEO_3188_ in vitreous ice. (B) Histogram of the membrane thickness of individual polymersomes made of PIPEO_3188_ based on the analysis of *N* = 65 vesicles.

Based on the capacitance measurements and an estimated membrane area of roughly 5000 µm^2^, the membrane thicknesses for PIPEO_877_/DPhPC, PIPEO_1530_/DPhPC and PIPEO_3188_/DPhPC were calculated to be 7.3 nm, 7.3 nm and 10.1 nm, respectively. These values were similar to the thickness of pure PIPEO_3188_ membranes in vitreous ice imaged by transmission electron cryo-microscopy (Figure 3).

### Channel activity of OmpF in lipopolymer membranes

**Figure 4:**
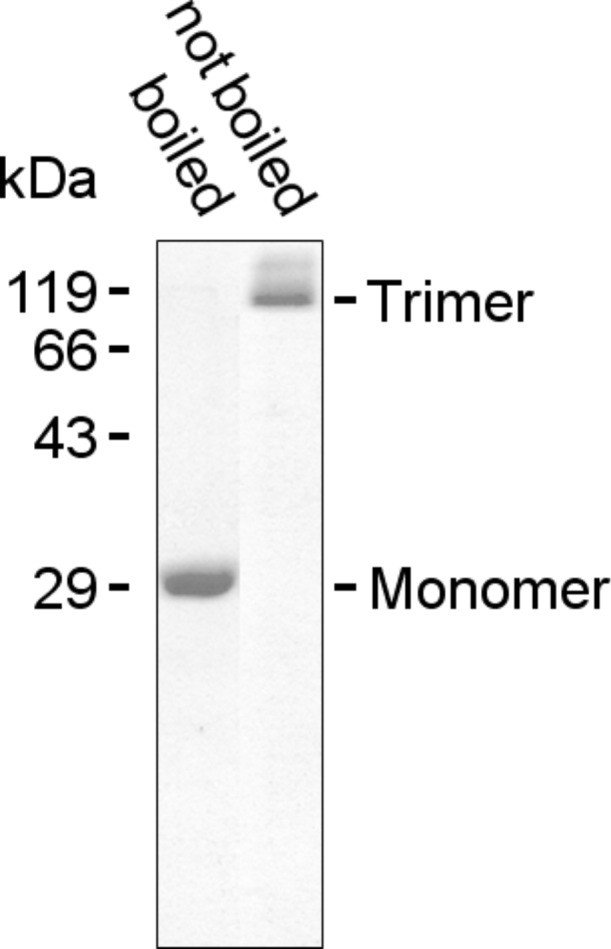
SDS-PAGE of OmpF. The protein was purified from *E. coli* membranes by differential extractions using SDS at temperatures of 50 and 37 °C, followed by dialysis against 1% octyl-POE. The monomeric and trimeric forms of OmpF were observed in boiled and unboiled samples, respectively.

Next, we studied the integration of OmpF (Figure 4) into planar PIPEO/DPhPC membranes of different thicknesses. With increasing molecular weight of PIPEO, the character of the formed assemblies was expected to change from more fluid and phospholipid-like to a thicker, stiffer polymeric behaviour [18,59], similar to membranes composed of poly(2-methyl-2-oxazoline-*block*-dimethylsiloxan-*block*-2-methyl-2-oxazoline) triblock copolymers [15,30,32].

**Figure 5:**
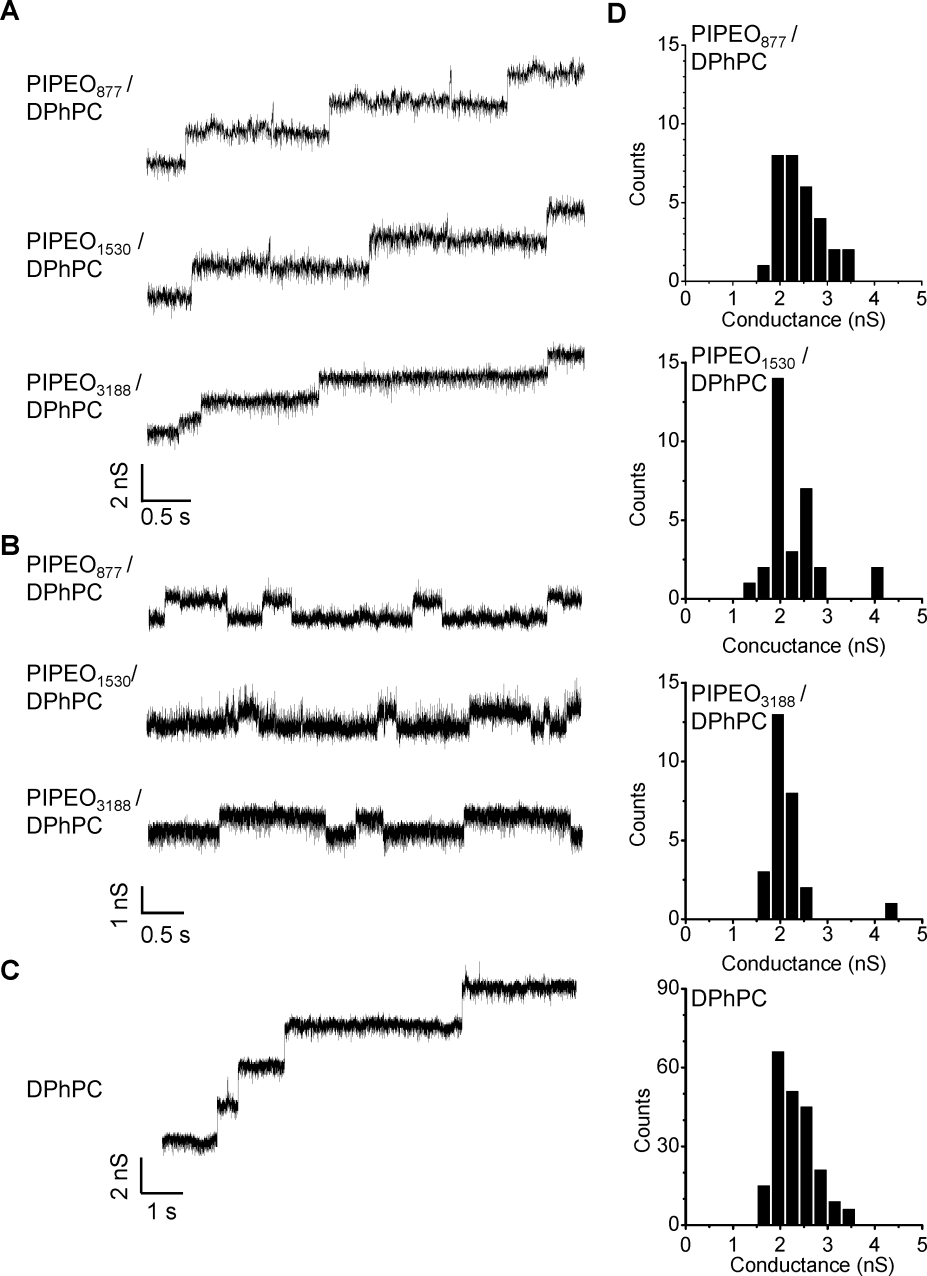
Channel activity of OmpF in planar lipopolymer membranes. Purified OmpF was added to both sides of the membrane formed of different PIPEO/DPhPC mixtures with polymer molar fraction of 0.9. A solution of 1 M KCl was applied as electrolyte. (A) Insertion of OmpF into the membranes. A membrane potential of Δ*V* = +50 mV (PIPEO877/10 mol % DPhPC and PIPEO1530/10 mol % DPhPC) and Δ*V* = +60 mV (PIPEO3188/10 mol % DPhPC) was applied. (B) Current traces of individual channels of integrated OmpF in lipopolymer membranes at a membrane potential of Δ*V* = +120 mV. (C) Current trace of OmpF channels integrating into a planar DPhPC membrane. The membrane voltage has been clamped to Δ*V* = −50 mV. (D) Amplitude histograms (bin width 0.3 nS) of channel conductance values of OmpF. Recording of conductance increments were carried out as described in (A) and (C). The data represent the conductance valuess of *N* = 31 (lipopolymer mixtures) and *N* = 213 (pure DPhPC) measurements, respectively.

Integration of OmpF into the membranes was monitored by measuring transmembrane ion currents (Figure 5A). Current recordings taken at a low membrane potential of 50 mV or 60 mV showed characteristic steps of current increase reflecting the insertion of OmpF into all membranes, independent of the molecular weight of the PIPEO present. At higher voltages, in particular above 100 mV, gating in the transmembrane current was observed for all lipopolymer membranes (Figure 5B). This was attributed to reversible closing and opening of individual channels of OmpF [60–62]. A total of 31 insertion events revealed an average conductance level of OmpF in the membranes of about 2.1 nS to 2.3 nS at low membrane potentials, as also demonstrated in the histograms (Figure 5D). At high membrane potentials, single-channel conductance was about 0.7 nS in 1 M KCl (Table 3), likely representing the conductance of a single pore of the OmpF trimer. These values were in line with conductance measurements of OmpF in pure DPhPC membranes in 1 M KCl (Figure 5C and Figure 5D) [60,62–63].

**Table 3:** Single-channel conductance values of the OmpF pore in lipopolymer membranes.

membrane composition	membrane potential (mV)	conductance in 1 M KCl^a^ (nS)

PIPEO_877_/DPhPC	50	2.3 ± 0.1
	120	0.7 ± 0.1
PIPEO_1530_/DPhPC	50	2.3 ± 0.1
	120	0.7 ± 0,1
PIPEO_3188_/DPhPC	60^b^	2.1 ± 0.1
	120	0.7 ± 0.1

^a^Mean ± s.e.m, *N* = 31; ^b^note that there was no insertion of OmpF into the membrane at a potential of +50 mV.

It should be noted that the probability of OmpF to insert into PIPEO/DPhPC membranes was significantly lower than that of inserting into planar DPhPC bilayers. A possible explanation is the presence of minute amounts of chloroform in the PIPEO/DPhPC membranes. Being well aware of the low compatibility of this solvent with proteins, this solvent led to the molecular dissolution of PIPEO. The presence of chloroform in our lipopolymer membranes may also be the reason why we did not observe typical three-step closing of trimeric OmpF channels at high voltages (Δ*V* > 100 mV).

Nevertheless, OmpF reconstituted in the lipopolymer membranes displayed typical voltage-dependent conductivities. This was in contrast to earlier studies on OmpF integrated into pure polymeric membranes composed of poly(2-methyl-2-oxazoline-*block*-dimethylsiloxan-*block*-2-methyl-2-oxazoline) triblock copolymer that showed no voltage-dependent closing and thus single-channel conductance for OmpF even at transmembrane voltages of 200 mV [38].

**Figure 6:**
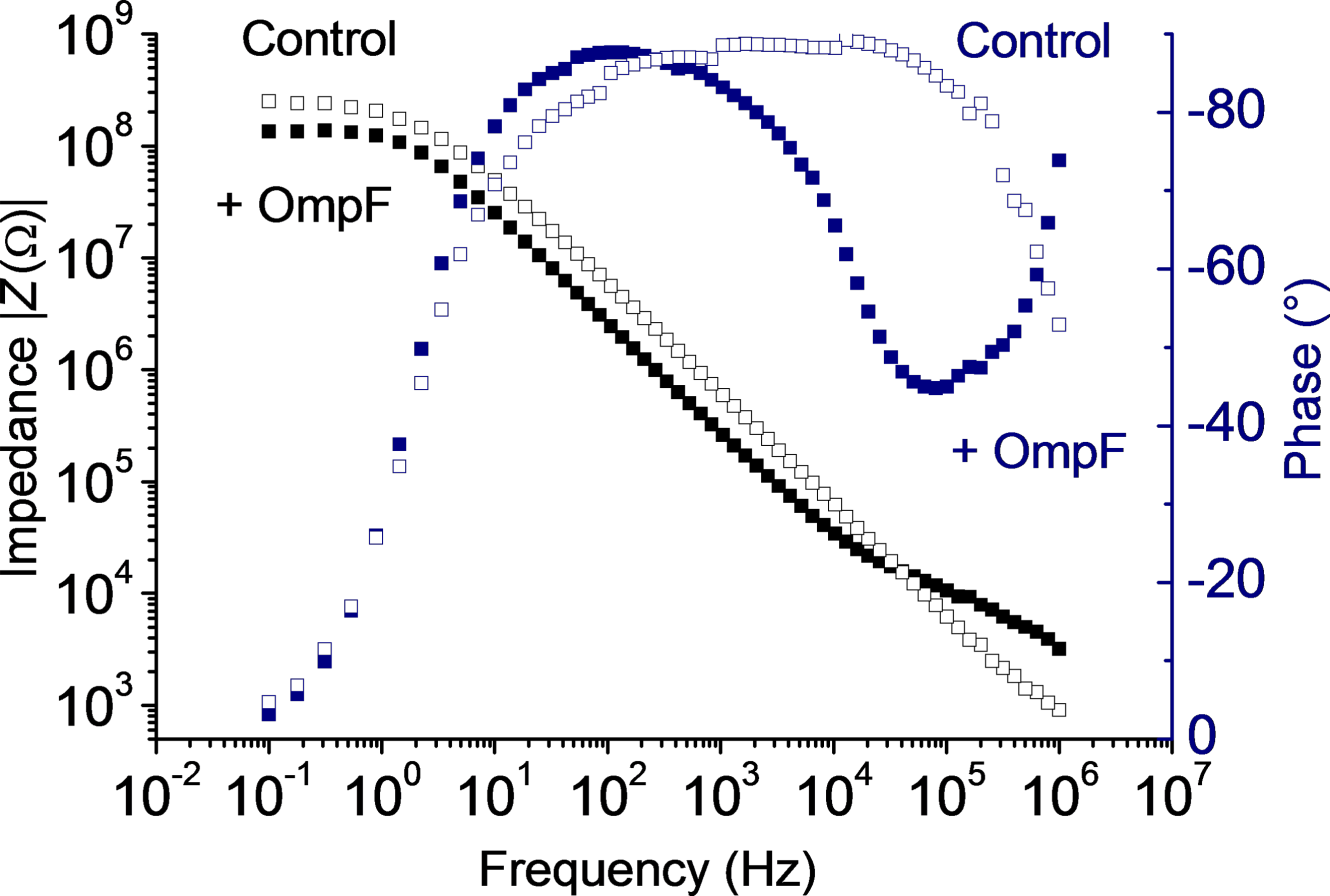
Bode representation of impedance and phase data of a planar PIPEO_1530_/DPhPC membrane in buffer containing 1 M KCl before and after incorporation of OmpF. The lowering of membrane resistance and the appearance of a second plateau-like feature at higher frequencies indicate the OmpF present in the membrane. |*Z*| in absence (empty squares) and presence (filled squares) of OmpF; phase in absence (empty circles) and presence (filled circles) of OmpF.

To provide further evidence of OmpF insertion into lipopolymer membranes, we also followed the integration of OmpF by using impedance spectroscopy. Figure 6 exemplarily shows an impedance spectrum of a PIPEO_1530_/DPhPC membrane in buffer containing 1 M KCl in absence and presence of OmpF. In the absence of OmpF, the |*Z*(Ω)| spectrum shows a frequency-independent plateau at low frequencies and a linear decrease with increasing frequencies. The monotonic decay of the impedance with frequency can be accounted for by the membrane capacitance, the plateau at low frequencies by the membrane resistance. This interpretation is supported by the detected phase shift that tended to be 0° at high frequencies, accounting for an ohmic resistance, whereas the 90° shift in parallel with the monotonic decay of impedance between 10 Hz and 10^5^ Hz accounted for a capacitive contribution. Upon integration of OmpF the spectrum changed. At the low-frequency end, lower levels of impedance were detected in parallel with a phase shift of 0°, indicating a reduced membrane resistance as expected for membranes perforated by protein pores [16]. At the high-frequency end of the spectrum, the shoulder in the impedance in parallel with the local minimum in the phase shift indicated a new ohmic element that also supports insertion of OmpF into the membrane [56].

According to the detected decrease in membrane resistance of approximately 100 MΩ and a trimeric conductance of OmpF of roughly 2 nS [60], the number of functional OmpF pores present in the membrane was calculated to be five. This demonstrates the sensitivity of impedance spectroscopy towards changes in the system to be analyzed [64]. Combined with the results for channel activity mentioned previously, the presence of functional OmpF in the lipopolymer membranes is strongly indicated, although no complete typical channel activity was observeable.

## Conclusion

In the past years, lipopolymer membranes gained more and more interest, as the properties of such assemblies can be steadily varied between the characteristics of a lipid bilayer and that of synthetic polymer membranes. By simply mixing lipids and amphiphilic polymers, a plethora of possible chemical and mechanical characteristics of polymeric membranes can be combined with the inherent biofunctionality of lipid bilayers [39–45,54]. Thereby, lipopolymer membranes became attractive also for membrane protein reconstitution. To our knowledge, the function of reconstituted proteins, however, has not been assessed in these systems yet.

Previous studies have shown that integration of membrane proteins into pure polymeric membranes depends more on the polymer flexibility than on the membrane thickness [15,24,31,65]. In this study, we selected amphiphilic PIPEO block copolymers and blended them with DPhPC to form lipopolymer membranes. Compared with other polymers used for the formation of biomimetic membranes [29–30,32], the hydrophobic PI blocks of PIPEO mimic the structure of unsaturated hydrophobic tails of lipids more closely, providing a more biomimetic character of the generated membranes. The obtained lipopolymer membranes exhibited a homogeneous distribution of its components and allowed for the reconstitution of OmpF as well as for a measurement of its channel activity. Until now, this could not be achieved for OmpF and has only rarely been assessed for other membrane proteins [29–30,38].

The enhanced mechanical stability of synthetic polymer membranes may expand the field of possible applications towards new technical processes using membrane proteins.

## Experimental

All chemicals used were of analytical grade and were obtained from Sigma Aldrich (Steinheim, Germany) unless stated otherwise. Isoprene was purchased from ABCR (Karlsruhe, Germany), ethylene oxide from Linde (Pullach, Germany).

### Polymer synthesis

Typical techniques for anionic polymerizations including high-vacuum techniques were used to synthesize the block copolymers as described for example in the review of Hadjichristidis et al. [66]. Solvent and monomers were purified prior to use. Toluene was stirred over Na–K alloy and benzophenone. Isoprene and ethylene oxide were purified in a two-step procedure: First, the monomers were stirred over calcium hydride for 1 h at −70 °C (dry ice–ethanol mixture), then transferred to a second flask and stirred for 1 h over di-*n*-butylmagnesium under the same conditions. To transfer the solvent and the monomers from one flask to another, cryo-distillation under static vacuum conditions was used.

The typical procedure for polymerization of the block copolymers is briefly mentioned here. To a volume of 100 mL of purified toluene in a 2-neck 250 mL reaction flask connected to the vacuum line, the desired volume (see Table 4) of purified isoprene was added at −140 °C. Just at the melting point of the isoprene solution, *sec*-butyllithium was added under an Ar atmosphere via syringe at 5 °C through a rubber septum in the quantity needed. Polymerization was carried out at 35 °C overnight. After polymerization of the isoprene block, a small sample of the precursor reaction mixture was collected via a syringe under an Ar atmosphere for analysis. The precursor was precipitated in cold ethanol as oil, decanted and dried in vacuum for further analysis.

**Table 4:** Used amounts of isoprene, ethylene oxide, *sec*-butyllithium and triisobutylaluminium for block copolymer synthesis.

block copolymer	isoprene (mL)	ethylene oxide (mL)	*sec*-butyllithium^a^ (mL)	triisobutylaluminium (mL)

PIPEO_877_	5	1.8	2.76	1.63
PIPEO_1530_	5	1.7	2.30	1.64
PIPEO_3188_	10	3.5	1.74	1.30

^a^Solution in cyclohexane (*c* = 1.4 M).

The sequential polymerization of ethylene oxide is a modification of the method published by Reijsek et al. [52]. Triisobutylaluminium was added to the reaction mixture at room temperature at the double stoichiometry of the active polyisoprenyl chains. After 3 h, the desired volume of purified ethylene oxide at −110 °C is distilled to the reaction mixture and polymerization of the second block is carried out at room temperature over night. Active chains are deactivated by adding a degassed 1:1 mixture of acetic acid and methanol via a syringe. After polymerization, the block copolymer is isolated by precipitation in cold ethanol, decanted, then re-dissolved in chloroform, filtrated and dried in vacuum. PIPEOs are stored at −20 °C under an Ar atmosphere.

### Polymer analysis

#### Size-exclusion chromatography

SEC was performed on a SECcurity System (PSS GmbH, Mainz, Germany) at 40 °C. Sample signals were detected with Agilent RI and UV-DAD detectors. THF was used as solvent with a flow rate of 0.5 mL min^−1^. Two SDV colums with 1000 Å pore size were used. Calibration of the system was done with poly(styrene) standards (ReadyCal, PSS GmbH, Mainz, Germany). Samples were evaluated with the PSS WinGPC Unichrom software, version 8.10. For analysis, approx. 5 mg of the polymer sample were dissolved in THF at a concentration between 2 mg mL^−1^ and 4 mg mL^−1^. A volume of 1 µL of *o*-dichlorobenzene was added to each sample as internal standard.

#### Nuclear magnetic resonance spectroscopy

Approx. 30 mg of sample was dissolved in 0.6 mL deuterated chloroform (Deutero, Kastellaun, Germany) and measured at 500 MHz with a Bruker Avance 500 spectrometer. The solvent residual peak in the spectrum was used as a reference for chemical shifts. The spectra were evaluated using the MestReNova Software.

#### Cryo transmission electron microscopy

To examine the morphology and membrane thickness of polymer vesicles by transmission electron microscopy (TEM), a drop of the vesicle solution of interest was placed on copper grids with lacey carbon film (200 mesh, Plano, Wetzlar, Germany), blotted with filter paper, vitrified in liquid ethane (Zeiss Cryobox, Zeiss NTS GmbH, Germany) and stored in liquid nitrogen until inserted under nitrogen atmosphere within a cryo transfer holder (CT3500, Gatan, USA) to the TEM column. Specimens were observed at a temperature of approx. −180 °C using a LEO922 OMEGA EFTEM (Zeiss, Oberkochen, Germany) operated at 200 kV.

### Block copolymer–phospholipid mixtures

Mixtures of block copolymers and phospholipids (molar ratio 9:1) were prepared by combining 200 µL of block copolymer solutions (10 mg mL^−1^ in THF) with solutions (2 mg mL^−1^ in chloroform) of a mixture (97.5:2.5 molar ratio) of 1,2-diphytanoyl-*sn*-glycero-3-phosphocholine (DPhPC) and 1-palmitoyl-2-(dipyrrometheneboron difluoride)undecanoyl-*sn*-glycero-3-phosphocholine (TopFluorPC, both Avanti Polar Lipids, Alabaster, USA). The block copolymer solutions of PIPEO_877_, PIPEO_1530_ and PIPEO_3188_ were mixed with 107.42, 61.57 and 29.55 µL of the phospholipid solution, respectively. From these mixtures, giant unilamellar vesicles (GUV) were formed using the electroformation technique [53]. For mixtures comprising of PIPEO_877_ and PIPEO_3188_, an alternating voltage of 3 V at 70 Hz was applied with the Vesicle Prep Pro (Nanion, Munich, Germany). Mixtures based on PIPEO_1530_ were subjected to an alternating voltage of 10 V at 300 Hz. As electrolyte, a saccharose solution (260 mM) was used.

To observe the mixing behavior of the lipopolymer mixtures, one day after vesicle formation, the GUV were observed with confocal fluorescence microscopy using a Zeiss LSM710 (Carl Zeiss, Oberkochen, Germany) at excitation wavelengths of 488 and 561 nm. Respectively, the emission of the TopFluorPC lipid was detected between 493 and 543 nm, and nile red emission in the range of 594 to 753 nm using a plan apo 63× oil immersion objective.

### Isolation of OmpF

Native OmpF protein was purified from *Escherichia coli* strain B^E^ BL21(DE3)omp6 [9], lacking both LamB and OmpC [6,10]. Briefly, cells from a one liter culture were resuspended in 50 mM Tris-HCl buffer (pH 7.5) containing 2 mM MgCl_2_ and DNAse and broken by passing through a French press. Unbroken cells were removed by a low-speed centrifugation, then, the supernatant was centrifuged at 100000*g* for 1 h. The pellet was resuspended in 50 mM Tris-HCl (pH 7.5) and mixed with an equal volume of SDS buffer containing 4% (w/v) sodium dodecyl sulfate (SDS), 2 mM β-mercaptoethanol and 50 mM Tris-HCl (pH 7.5). After 30 min incubation at a temperature of 50 °C, the solution was centrifuged at 100000*g* for 1 h. The pellet was resuspended in SDS-salt buffer containing 2% SDS, 0.5 M NaCl, 50 mM Tris-HCl (pH 7.5), incubated at a temperature of 37 °C for 30 min and centrifuged again at 100000*g* for 30 min. The supernatant containing OmpF was dialysed overnight against 20 mM Tris (pH 8), 1 mM EDTA and 1% (w/v) *n*-octyl polyoxyethylene (octyl-POE). The purity of the protein was assessed by SDS-PAGE.

### Formation of planar lipopolymer membranes

Planar membranes of the lipopolymer mixtures were prepared across a circular aperture with a diameter of 400 µm in the partitioning wall of a custom-built black lipid bilayer Teflon chamber. The volume of each compartment, separated by the partionining wall, was 5 mL [67]. PIPEO/DPhPC in chloroform (10 µL) was applied around the aperture of the chamber and allowed to dry to get rid of organic solvent. Then, the chambers on both sides of the aperture were filled with degassed KCl saline (1 M KCl, 20 mM Tris (pH 8)) and 2 µL of PIPEO/DPhPC in chloroform was painted across the aperture with a fine brush. Successful self-assembly of a membrane was monitored by capacitance measurements.

### Electrochemical impedance spectroscopy

Electrochemical impedance spectroscopy of PIPEO, PIPEO/DPhPC and DPhPC membranes was performed using a Zahner IM6 electrochemical workstation equipped with HighZ-Probes (Zahner-Elektrik, Kronach, Germany), respectively. The HighZ-Probes were connected to Ag/AgCl electrodes immersed into the electrolyte compartments on both sides of the formed membranes. Measurements were performed in the range between 1 MHz and 100 mHz at an offset of 0 mV and an amplitude of 10 mV. Data acquisition was performed with the Thales Z-Man 1.18 software package from Zahner-Elektrik. The set-up was covered by a custom-built Faraday cage.

### Single- and multi-channel measurements

Current fluctuations through single and multiple OmpF channels reconstituted into planar PIPEO/DPhPC and DPhPC bilayers were recorded following standard protocols [9,68–69]. After formation of the bilayers we made sure, that the current trace reflects a stable baseline. Then OmpF was added by pipetting 10 to 50 µL of purified protein (1 mg mL^−1^ to 2 mg mL^−1^ in 20 mM Tris (pH 8), 1 mM EDTA and 1% w/v octyl-POE) to both sides of the membrane. At this dilution, the concentration of octyl-POE (0.01% w/v) is below its cmc value and a channel-like structure, formed by the surfactant is very unlikely. Current fluctuations through single and multiple channels were recorded in voltage-clamp mode using an Axon Axopatch 200B amplifier (Molecular Devices, Sunnyvale, USA) with a capacitive headstage (Axon CV203 BU) connected to the bilayer chambers by a pair of custom-made Ag/AgCl electrodes. Current signals were filtered at 2 kHz and digitally sampled at 10 kHz with an Axon Digidata 1322A digitizer controlled by the Clampex 10.2 software (Molecular Devices, Sunnyvale, USA).

## Supporting Information

File 1 Additional experimental data.

## References

[1] Branton D, Deamer D W, Marziali A, Bayley H, Benner S A, Butler T, Di Ventra M, Garaj S, Hibbs A, Huang X (2008). Nat Biotechnol.

[2] Derrington I M, Butler T Z, Collins M D, Manrao E, Pavlenok M, Niederweis M, Gundlach J H (2010). Proc Natl Acad Sci U S A.

[3] Loman N J, Quick J, Simpson J T (2015). Nat Methods.

[4] Loman N J, Watson M (2015). Nat Methods.

[5] Ip C L C, Loose M, Tyson J R, de Cesare M, Brown B L, Jain M, Leggett R M, Eccles D A, Zalunin V, Urban J M (2015). F1000Research.

[6] Kusters I, van den Bogaart G, Kedrov A, Krasnikov V, Fulyani F, Poolman B, Driessen A J (2011). Structure.

[7] Mahendran K R, Lamichhane U, Romero-Ruiz M, Nussberger S, Winterhalter M (2013). J Phys Chem Lett.

[8] Mahendran K R, Romero-Ruiz M, Schlösinger A, Winterhalter M, Nussberger S (2012). Biophys J.

[9] Romero-Ruiz M, Mahendran K R, Eckert R, Winterhalter M, Nussberger S (2010). Biophys J.

[10] Kowalczyk S W, Kapinos L, Blosser T R, Magalhães T, van Nies P, Lim R Y, Dekker C (2011). Nat Nanotechnol.

[11] Wang H, Chung T-S, Tong Y W, Jeyaseelan K, Armugam A, Chen Z, Hong M, Meier W (2012). Small.

[12] Zhao Y, Qiu C, Li X, Vararattanavech A, Shen W, Torres J, Hélix-Nielsen C, Wang R, Hu X, Fane A G (2012). J Membr Sci.

[13] Zhong P S, Chung T-S, Jeyaseelan K, Armugam A (2012). J Membr Sci.

[14] Langowska K, Palivan C G, Meier W (2013). Chem Commun.

[15] Palivan C G, Goers R, Najer A, Zhang X, Car A, Meier W (2015). Chem Soc Rev.

[16] Schmitt E K, Steinem C, Eliaz N (2012). Electrochemical Analysis of Ion Channels and Transporters in Pore-Suspending Membranes. Applications of Electrochemistry and Nanotechnology in Biology and Medicine II.

[17] Yuan H, Leitmannova-Ottova A, Tien H T (1996). Mater Sci Eng, C.

[18] Discher B M, Won Y-Y, Ege D S, Lee J C-M, Bates F S, Discher D E, Hammer D A (1999). Science.

[19] Förster S, Plantenberg T (2002). Angew Chem, Int Ed.

[20] Antonietti M, Förster S (2003). Adv Mater.

[21] Ahmed F, Photos P J, Discher D E (2006). Drug Dev Res.

[22] Dimova R, Seifert U, Pouligny B, Förster S, Döbereiner H-G (2002). Eur Phys J E.

[23] Itel F, Chami M, Najer A, Lörcher S, Wu D, Dinu I A, Meier W (2014). Macromolecules.

[24] Mecke A, Dittrich C, Meier W (2006). Soft Matter.

[25] Lin J J, Bates F S, Hammer D A, Silas J A (2005). Phys Rev Lett.

[26] Lin J J, Ghoroghchian P P, Zhang Y, Hammer D A (2006). Langmuir.

[27] Lin J J, Silas J A, Bermudez H, Milam V T, Bates F S, Hammer D A (2004). Langmuir.

[28] Domes S, Filiz V, Nitsche J, Frömsdorf A, Förster S (2010). Langmuir.

[29] Shen Y-x, Saboe P O, Sines I T, Erbakan M, Kumar M (2014). J Membr Sci.

[30] Zhang X, Tanner P, Graff A, Palivan C G, Meier W (2012). J Polym Sci, Part A: Polym Chem.

[31] Pata V, Dan N (2003). Biophys J.

[32] Malinova V, Belegrinou S, de Bruyn Ouboter D, Meier W P (2010). Adv Polym Sci.

[33] Lee A G (2004). Biochim Biophys Acta, Biomembr.

[34] Phillips R, Ursell T, Wiggins P, Sens P (2009). Nature.

[35] Laganowsky A, Reading E, Allison T M, Ulmschneider M B, Degiacomi M T, Baldwin A J, Robinson C V (2014). Nature.

[36] Chen F-Y, Lee M-T, Huang H W (2002). Biophys J.

[37] Keller S L, Bezrukov S M, Gruner S M, Tate M W, Vodyanoy I, Parsegian V A (1993). Biophys J.

[38] Meier W, Nardin C, Winterhalter M (2000). Angew Chem, Int Ed.

[39] Le Meins J-F, Schatz C, Lecommandoux S, Sandre O (2013). Mater Today.

[40] Le Meins J-F, Sandre O, Lecommandoux S (2011). Eur Phys J E.

[41] Chemin M, Brun P-M, Lecommandoux S, Sandre O, Le Meins J-F (2012). Soft Matter.

[42] Nam J, Beales P A, Vanderlick T K (2011). Langmuir.

[43] Schulz M, Glatte D, Meister A, Scholtysek P, Kerth A, Blume A, Bacia K, Binder W H (2011). Soft Matter.

[44] Schulz M, Olubummo A, Bacia K, Binder W H (2014). Soft Matter.

[45] Nam J, Vanderlick T K, Beales P A (2012). Soft Matter.

[46] Kowal J, Wu D, Mikhalevich V, Palivan C G, Meier W (2015). Langmuir.

[47] Thoma J, Belegrinou S, Rossbach P, Grzelakowski M, Kita-Tokarczyk K, Meier W (2012). Chem Commun.

[48] Watanabe Y (2002). J Protein Chem.

[49] Watanabe Y (2002). J Chromatogr A.

[50] Phale P S, Philippsen A, Kiefhaber T, Koebnik R, Phale V P, Schirmer T, Rosenbusch J P (1998). Biochemistry.

[51] Discher D E, Eisenberg A (2002). Science.

[52] Rejsek V, Desbois P, Deffieux A, Carlotti S (2010). Polymer.

[53] Angelova M I, Soléau S, Méléard P, Faucon F, Bothorel P, Helm C, Lösche M, Möhwald H (1992). Preparation of giant vesicles by external AC electric fields. Kinetics and applications. Trends in Colloid and Interface Science VI.

[54] Dao T P T, Fernandes F, Er-Rafik M, Salva R, Schmutz M, Brûlet A, Prieto M, Sandre O, Le Meins J-F (2015). ACS Macro Lett.

[55] Schulz M, Werner S, Bacia K, Binder W H (2013). Angew Chem, Int Ed.

[56] Guidelli R, Becucci L, Eliaz N (2012). Electrochemistry of Biomimetic Membranes. Applications of Electrochemistry and Nanotechnology in Biology and Medicine II.

[57] Schmitt E K, Weichbrodt C, Steinem C (2009). Soft Matter.

[58] Steinem C, Janshoff A, Galla H-J, Sieber M (1997). Bioelectrochem Bioenerg.

[59] Aranda-Espinoza H, Bermudez H, Bates F S, Discher D E (2001). Phys Rev Lett.

[60] Benz R (1985). Crit Rev Biochem.

[61] Danelon C, Suenaga A, Winterhalter M, Yamato I (2003). Biophys Chem.

[62] Schmitt E K, Vrouenraets M, Steinem C (2006). Biophys J.

[63] Benz R, Janko K, Boos W, Läuger P (1978). Biochim Biophys Acta, Biomembr.

[64] Barsoukov E, Macdonald J R (2005). Impedance spectroscopy: theory, experiment, and applications.

[65] Itel F, Najer A, Palivan C G, Meier W (2015). Nano Lett.

[66] Hadjichristidis N, Iatrou H, Pispas S, Pitsikalis M (2000). J Polym Sci, Part A: Polym Chem.

[67] Arnold T, Poynor M, Nussberger S, Lupas A N, Linke D (2007). J Mol Biol.

[68] Mager F, Gessmann D, Nussberger S, Zeth K (2011). J Membr Biol.

[69] Poynor M, Eckert R, Nussberger S (2008). Biophys J.

