# Supplementary material for: Reconstitution of the membrane protein OmpF into biomimetic block copolymer–phospholipid hybrid membranes
Source: Beilstein J Nanotechnol. 2016 Jun 21;7:881–92. doi: 10.3762/bjnano.7.80 (PMC4979867; doi:10.3762/bjnano.7.80)
Supplement: File 1 — Additional experimental data. [file Beilstein_J_Nanotechnol-07-881-s001.pdf]

Supporting Information

for

Reconstitution of the membrane protein OmpF into biomimetic block copolymer–phospholipid hybrid membranes

Matthias Bieligmeyer[1,§], Franjo Artukovic[2], Stephan Nussberger*[2], Thomas Hirth[1,3], Thomas Schiestel[3] and Michaela Müller*[3]

Address: [1]Institute of Interfacial Process Engineering and Plasma Technology, Department of Chemical Interfacial Process Engineering, University of Stuttgart, Nobelstraße 12, 70569 Stuttgart, Germany; [2]Institute of Biomaterials and Biomolecular Systems, Department of Biophysics, University of Stuttgart, Pfaffenwaldring 57, 70550 Stuttgart, Germany and [3]Fraunhofer Institute for Interfacial Engineering and Biotechnology, Department of Interfacial Engineering and Materials Science, Nobelstraße 12, 70569 Stuttgart, Germany

Email: Michaela Müller* - Michaela.Mueller@igb.fraunhofer.de;
Stephan Nussberger* - Nussberger@bio.uni-stuttgart.de

*Corresponding author

[§]Current address: Aesculap AG, Am Aesculap-Platz, 78532 Tuttlingen, Germany

Additional experimental data

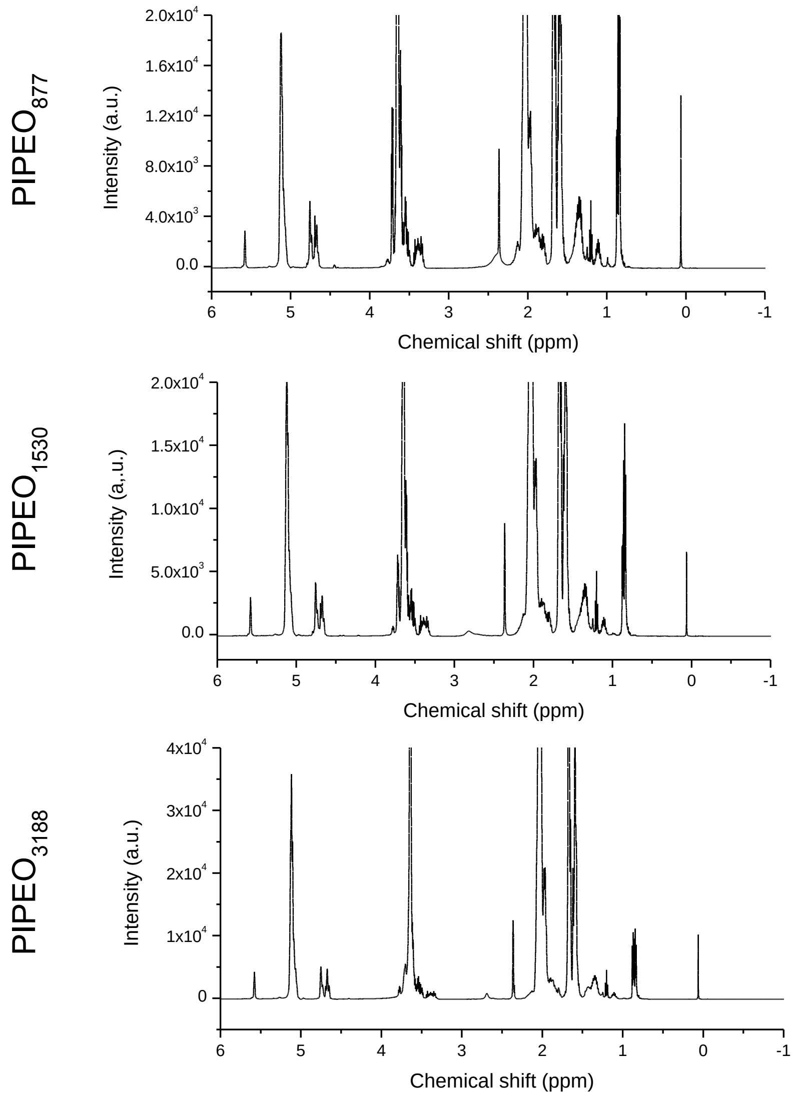

Figure S1: ^{1}H NMR spectra of synthesized PIPEO block copolymers. Characteristic signals for this type of block copolymer are the ones at 5.6, 5.1, 4.8 and 3.6 ppm. The latter one represents the signal coming from the CH_2-groups in the PEO repeating units, whereas the other signals come from the =C-H or =CH_2 groups within the isoprene repeating units. The amount of 1,4-isoprene repeating units has been determined by comparing the integrals of the signals at 5.6, 5.1and 4.8 ppm. It must be noted here, that the signal at 5.1 ppm is due to one proton within a 1,4-repeating unit, whereas the other two isoprene signals are due to two protons each in 1,2-repeating units. The signals with chemical shifts between 3 and 1 ppm are due to proton resonance within the polymer backbone. The signal coming from the *sec*-buthyl lithium residue of the initiator is observable at 0.85 ppm. Note, peak intensities were truncated, if greater than 2.0 × 10^4 a.u. and 4.0 × 10^4 a.u., respectively.

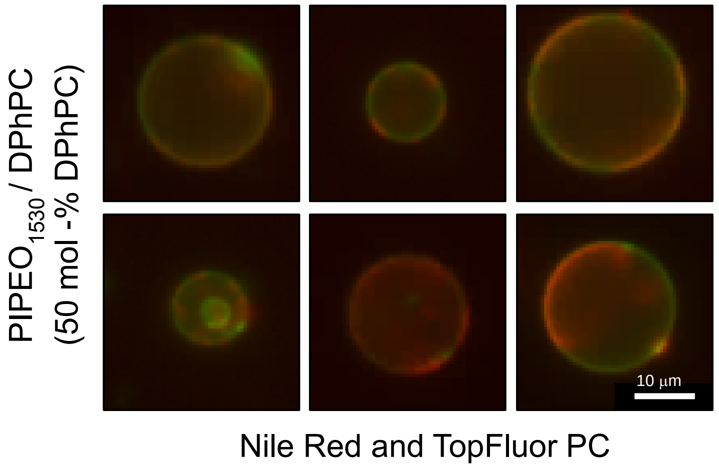

Figure S2: Optical micrographs of lipopolymersome formed of $PIPEO_{1530}$ and 50 mol % DPhPC. Staining membranes with the dye Nile Red (red) and the fluorescent lipid TopFluorPC (green) reveal a heterogeneous distribution of both dyes indicating phase separation of PIPEO and DPhPC.

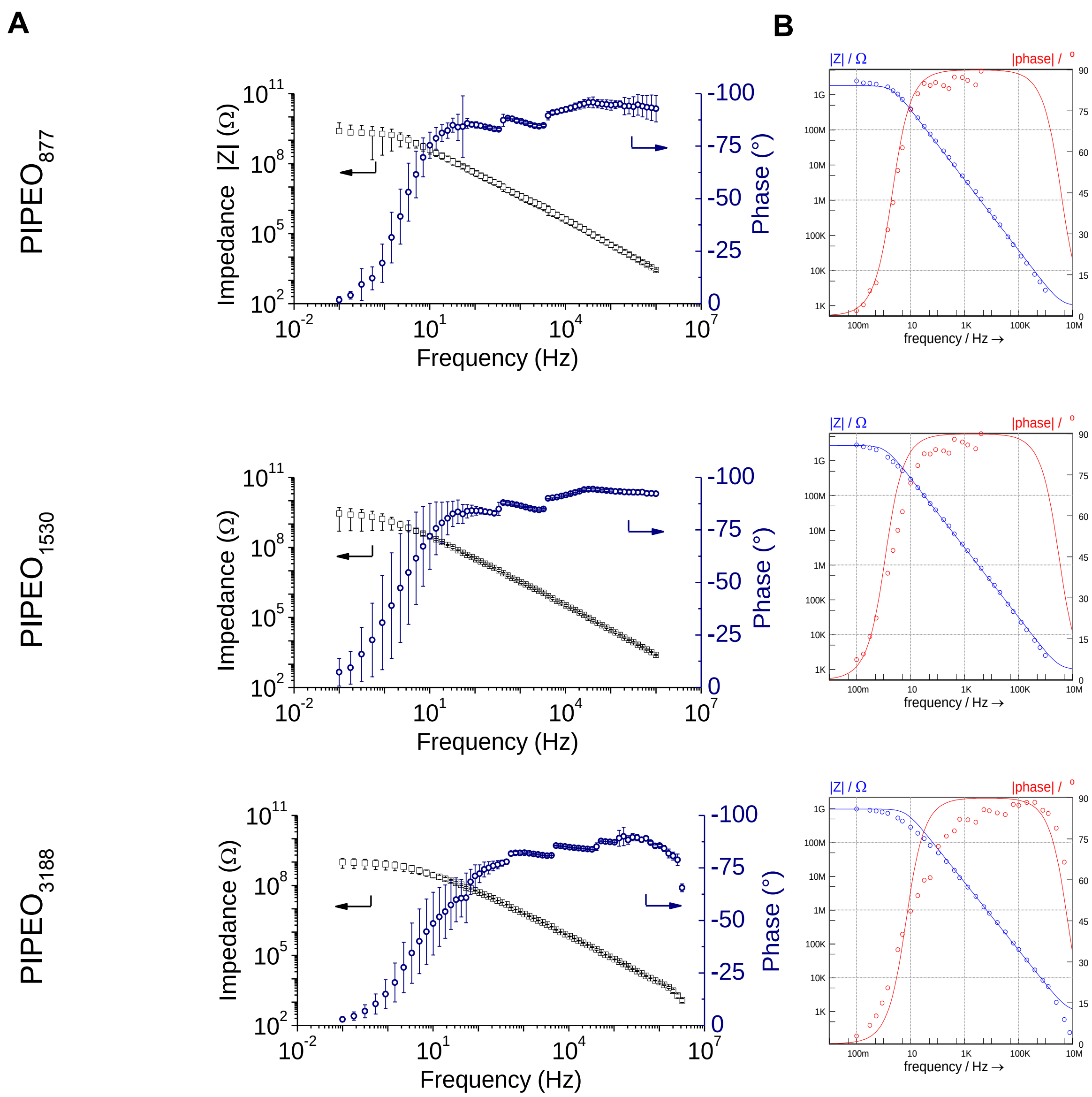

Figure S3: Analysis of the electrical properties of planar PIPEO polymer membranes based on impedance spectroscopy. (A) Bode representation of impedance |*Z*| (□, black) and phase (O, navy blue) data of planar $PIPEO_{877}$, $PIPEO_{1530}$ and $PIPEO_{3188}$ membranes in buffer containing 1 M KCl, respectively. (B) Fit of the impedance data shown in (A) based on equivalent circuits consisting of an ohmic resistance in series with a resistance–capacitance (RC) pair in parallel, corresponding to an electrolyte–membrane–electrolyte interface.
